# Regulation of excitation-contraction coupling in mouse cardiac myocytes: integrative analysis with mathematical modelling

**DOI:** 10.1186/1472-6793-9-16

**Published:** 2009-08-31

**Authors:** Jussi T Koivumäki, Topi Korhonen, Jouni Takalo, Matti Weckström, Pasi Tavi

**Affiliations:** 1 Department of Physics, University of Oulu & Biocenter Oulu, Finland; 2 Department of Biotechnology and Molecular Medicine, A.I. Virtanen Institute for Molecular Sciences, University of Kuopio, Kuopio, Finland

## Abstract

**Background:**

The cardiomyocyte is a prime example of inherently complex biological system with inter- and cross-connected feedback loops in signalling, forming the basic properties of intracellular homeostasis. Functional properties of cells and tissues have been studied e.g. with powerful tools of genetic engineering, combined with extensive experimentation. While this approach provides accurate information about the physiology at the endpoint, complementary methods, such as mathematical modelling, can provide more detailed information about the processes that have lead to the endpoint phenotype.

**Results:**

In order to gain novel mechanistic information of the excitation-contraction coupling in normal myocytes and to analyze sophisticated genetically engineered heart models, we have built a mathematical model of a mouse ventricular myocyte. In addition to the fundamental components of membrane excitation, calcium signalling and contraction, our integrated model includes the calcium-calmodulin-dependent enzyme cascade and the regulation it imposes on the proteins involved in excitation-contraction coupling. With the model, we investigate the effects of three genetic modifications that interfere with calcium signalling: 1) ablation of phospholamban, 2) disruption of the regulation of L-type calcium channels by calcium-calmodulin-dependent kinase II (CaMK) and 3) overexpression of CaMK. We show that the key features of the experimental phenotypes involve physiological compensatory and autoregulatory mechanisms that bring the system to a state closer to the original wild-type phenotype in all transgenic models. A drastic phenotype was found when the genetic modification disrupts the regulatory signalling system itself, i.e. the CaMK overexpression model.

**Conclusion:**

The novel features of the presented cardiomyocyte model enable accurate description of excitation-contraction coupling. The model is thus an applicable tool for further studies of both normal and defective cellular physiology. We propose that integrative modelling as in the present work is a valuable complement to experiments in understanding the causality within complex biological systems such as cardiac myocytes.

## Background

The function of the mammalian heart depends essentially on the properties of individual heart muscle cells, i.e. cardiac myocytes. The complex chain of events that links action potential (AP) with contraction, known as excitation-contraction (E-C) coupling (for review see [1]), starts with myocyte membrane depolarization followed by opening of the voltage-gated L-type calcium channels (LCC) during the AP. This results in a Ca^2+ ^influx through the sarcolemma, which activates Ca^2+ ^release channels (the ryanodine receptors; RyRs) located in the junctional face of the sarcoplasmic reticulum (SR), and thereby induces calcium release from the SR (the calcium-induced calcium release; CICR). The resulting transient increase of the myoplasmic [Ca^2+^]_i _allows calcium binding to proteins of the contractile element that generate force and movement by utilizing the energy stored in the high-energy phosphate bonds of ATP. In order to avoid cumulative gain of Ca^2+ ^in the cytosol and for relaxation to occur, SR Ca^2+^-ATPase (SERCA) pumps Ca^2+ ^ions back into the SR to be released upon the next excitation, while the electrogenic Na^+^/Ca^2+^-exchanger (NCX) transports part of the released Ca^2+ ^out of the cell.

The dynamic regulation of the calcium transport mechanisms is essential at varying heart beating rates. To fulfil this task, the same cellular calcium signals that control the contraction are also decoded by calcium-dependent enzymes, like the Ca^2+^-calmodulin dependent kinases and phosphatases. These enzymes regulate the proteins involved in E-C coupling, providing physiologically important feedback mechanisms [2]. Since the same enzymes regulate nuclear gene expression during long term alterations in the calcium signals, they thus regulate differentiation, growth, metabolic and functional specializations, and eventually shape the phenotype of the muscle cells during development and pathogenesis [3,4]. A line of evidence suggests that the causes of heart failure involve defects in the regulation of cellular calcium and a concomitant reduction in cardiomyocyte contraction [5]. To elucidate the E-C coupling processes involved in these pathological developments, a number of genetically engineered mouse models have been generated and studied experimentally. These models include, among others, genetic ablation or overexpression of the E-C coupling machinery proteins [6,7] as well as transgenic overexpression of the regulative calcium activated enzymes [8,9]. The effects genetic interventions have on the function of the mouse E-C coupling machinery have been evaluated on the basis of the analysis of the phenotypes of the animals bearing the genetic modifications. These analyses have included measurements of morphological, histological and biochemical variables from mice hearts, and also biophysical and physiological analysis of the E-C coupling at the level of individual myocytes. This approach delivers precise data about the physiology at the endpoint, but in order to elucidate the processes that have lead to the endpoint phenotype it might be beneficial to use complementary tools, such as mathematical modelling.

To gain more mechanistic information from the signalling networks involved in cardiac E-C coupling and from the sophisticated genetically engineered mouse models, we have built a mathematical model of mouse cardiomyocyte function. In addition to the fundamental components of E-C coupling, contraction and membrane excitability, this integrated model also includes the Ca^2+^-calmodulin-dependent kinase II (CaMK) cascade and its modulatory effects on the proteins involved in E-C coupling. CaMK regulates the activity of ryanodine receptors [10], SERCA [11] and its regulative protein PLB [12,13] and L-type Ca^2+^-channels [14,15]. Because CaMK is activated by an increase in the cytosolic [Ca^2+^]_i _and, specifically, by the frequency of intracellular calcium transients [16,17], it constitutes a physiological feedback mechanism adjusting calcium signals to the current heart rate [18]. Importantly, although the presented model is highly simplified, it is complex enough to be robust and to have features that arise from the signalling networks rather than from the properties of individual components.

The developed model simulates the dynamic behaviour of cardiomyocytes with beating frequency-dependent adaptations in function of the E-C coupling proteins as one of the fundamental physiological features. It can be argued that the model is thus an applicable and powerful tool for the reproduction of genetic cardiomyocyte modifications and analysis of the resulting cellular signalling changes *in silico*. To establish this, we have used the model to mathematically produce three different genetically modified mouse cardiac myocytes: 1) cardiomyocytes with deleted SERCA inhibitory protein phospholamban (PLB) [19], 2) disruption of the CaMK-dependent regulation of LCC, and 3) heart failure model induced by overexpression of CaMK [20]. Our results not only reveal unexpected changes in cardiomyocyte function upon changes of the functions of single proteins, but also demonstrate that mathematical modelling is an essential tool in designing genetically modified mice models as well as exploring the cellular signalling cascades of the existing models.

## Results

### Features and Validation of the Model

**Figure 1:**
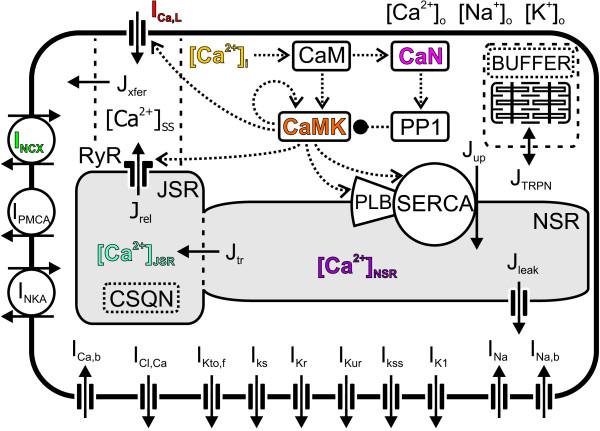
**Schematic presentation of the components and features of the model**. Model accounts for processes that regulate intracellular concentration changes of sodium, potassium and calcium ions. The Ca^2+ ^transport mechanisms are the L-type Ca^2+ ^current (I_Ca, L_), ryanodine receptor (RyR), SR Ca^2+ ^ATPase (SERCA), sarcolemmal Na^+^/Ca^2+ ^exchanger (NCX), sarcolemmal Ca^2+ ^ATPase (PMCA), and background Ca^2+ ^current (I_Ca, b_). The Ca^2+ ^fluxes within the cell are the uptake of Ca^2+ ^(J_up_) from the cytosol to the network sarcoplasmic reticulum (NSR), Ca^2+ ^release (J_rel_) from the junctional SR (JSR), Ca^2+ ^flux (J_tr_) from the NSR to JSR, Ca^2+ ^leak (J_leak_) from the NSR to the cytosol, Ca^2+ ^flux from the subspace (SS) volume to the bulk myoplasm (J_xfer_) and from the cytosol to Troponin (J_TRPN_). The cytosolic bulk Ca^2+ ^concentration is [Ca^2+^]_i_. The calcium concentrations in the SS, JSR and NSR compartments are [Ca^2+^]_SS_, [Ca^2+^]_JSR _and [Ca^2+^]_NSR_, respectively. The Ca^2+ ^buffers that operate in the JSR and related to TRPN are presented as CSQN and BUFFER, respectively. The input for the enzyme reactions is the intracellular cytosolic Ca^2+ ^concentration, [Ca^2+^]_i_. A rise in the [Ca^2+^]_i _level increases Ca^2+ ^binding to calmodulin (CaM), which in turn phosphorylates more Ca^2+^/calmodulin-dependent protein kinase II (CaMK) and calcineurin (CaN). Phosphorylation of the latter induces phosphorylation of protein phosphatase 1 (PP1). The autophosphorylation of CaMK is presented as a positive feedback loop and PP1 inhibition as a negative feedback. The model also includes the following transmembrane currents: the Ca^2+^-activated chloride (Cl^-^) current (I_Cl, Ca_), the rapidly recovering transient outward K^+ ^current (I_Kto, f_), the slow delayed rectifier K^+ ^current (I_Ks_), the rapid delayed rectifier K^+ ^current (I_Kr_), the ultrarapidly activating delayed rectifier K^+ ^current (I_Kur_), noninactivating steady-state voltage activated K^+ ^current (I_Kss_), the time-independent K^+ ^current (I_K1_), fast Na^+ ^current (I_Na_), Na^+ ^background current (I_Na, b_), and the Na^+^/K^+ ^pump (I_NKA_).

**Figure 2:**
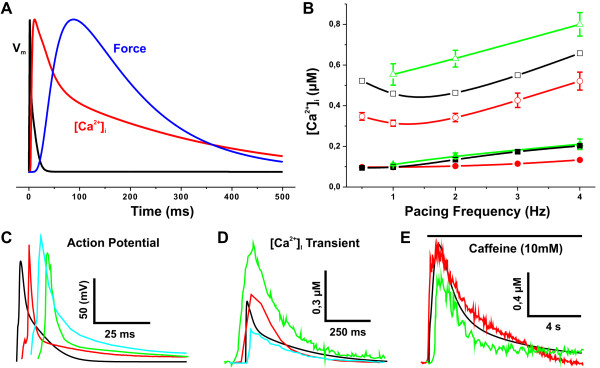
**Validation of the model characteristics**. (A) Normalized action potential (V_m_), [Ca^2+^]_i _transient and force amplitude at 1 Hz pacing. (B) Diastolic (closed symbols) and systolic (open symbols) [Ca^2+^]_i _values as a function of pacing frequency. Simulated values (black squares) are compared with the experimental results of Ito et al. [21] (red circles) and Antoons et al. [57](green triangles). The model was able to successfully reproduce the experimentally observed minimum in the systolic [Ca^2+^]_i _around 1-2 Hz [21]. (C) Simulated (black) action potential (AP) is compared to measurements by Guo et al. [58] (red), Brunet et al. [59] (green) and Petrashevskaya et al. [60] (cyan). The comparison of recorded and simulated APs (note that the APs have been shifted in time for comparison) demonstrates a good qualitative agreement with the experiments. (D) [Ca^2+^]_i _transient at 1 Hz pacing. Comparison of simulated results (black) to the experimental data of Williams et al. [61] (red), Maeir et al. [20] (green) and Huser et al. [32] (cyan) demonstrates a good qualitative agreement of *in silico *and largely variated *in vivo *data. (E) Caffeine-induced [Ca^2+^]_i _transients. The amplitude and decay time constants of simulated data (black) are of the same magnitude with the *in vivo *results of Maeir et al. [20] (red) and Li et al. [19] (green).

Our goal was to develop (see Methods section for details) a modelling tool with which we could simulate the key physiological aspects of the cardiac myocytes (Figure 1). The signals that the model produces are in line with the parameters from the experiments from mouse cardiac myocytes. Action potential features as well as resting potential values correspond to those reported previously (Table 1). Also, the model has accurate relative contributions of different calcium transport mechanisms that cooperatively generate calcium signals with parameters like diastolic and systolic [Ca^2+^]_i _as well as decays of calcium transients corresponding to the measured average values from various sources (Table 2). The variation among the published experimental results is surprisingly large (Figure 2B and 2C, and Table 2), probably due to differences in the experimentation and treatment of the cardiomyocytes. Therefore, it is not reasonable to tune the model parameters exactly to any single set of experimental results. More importantly, in order to verify the model we focused on the physiological behaviour of the variables and their trends during the intervention experiments. As an example of this, the model could successfully predict the behaviour of the experimentally observed diastolic [21-23] and systolic [Ca^2+^]_i _around 1-4 Hz (Figure 2B). In the experiments, a commonly used intervention to study the capacity of the intracellular calcium stores is the caffeine pulse experiment, where rapid application of caffeine empties the SR abruptly and produces a cytosolic calcium rise, the height of which corresponds to the amount of calcium stored in the SR. During the caffeine pulse, the calcium extrusion depends mainly on the NCX, and therefore the decay of the [Ca^2+^]_i _can be used to estimate the amount and activity of NCX in the given cell. We simulated a caffeine pulse experiment and compared the results to the *in vivo *data of Maier et al. [20] and Li et al. [19]. The amplitude of the simulated caffeine-induced intracellular calcium transient agreed well with the measurements. Also the decay constants of [Ca^2+^]_i _are of the same magnitude, 2.5 ± 0.3 s, 2.2 ± 0.2 s, and 1.8 ± 0.03 s for [20,19] and the model simulations, respectively. Therefore, the model fulfilled the criteria of being capable of reproducing real physiological phenomena, like the [Ca^2+^]_i_-frequency curve, and could robustly simulate the caffeine pulse experiment.

**Table 1:** Comparison of the AP properties of the model and experimental data

Magnitude	Simulated value	Experimental value
Quiescent [Na^+^]_i _(mM)	13.6	16.4 ± 0.7 [66]; 15.6 ± 0.7 [66]; 12.5 ± 1.8 [67]
Quiescent [K^+^]_i _(mM)	143.0	~140 [81]
Quiescent E_K _(mV)	-84.1	~-80 [81]
Quiescent V (mV)	-79.0	~E_K _[81]
AP amplitude (mV) at 1 Hz	110.6	106 ± 11 [20]; 118 [68]
AP 50% duration (ms) at 1 Hz	3.25	4.5 ± 0.3 [69]; 2.6 ± 0.1 [59]; 1.6 ± 0.2 [20]
dV/dt_max _(V/s) at 1 Hz	148.4	161 ± 44 (neonatal cell culture) [70] 193.25 ± 13.94 (rat) [71]; 133.8 ± 9.2 (42 days old) [58]

**Table 2:** Comparison of Ca^2+ ^handling properties of E-C coupling in the developed model and experiments

Magnitude	Simulated value	Experimental value
Diastolic [Ca^2+^]_i _(μM)	0.10	0.100 ± 0.012 [66]; 0.099 ± 0.011 [21]; 0.200 ± 0.021 [20]
Systolic [Ca^2+^]_i _(μM)	0.46	0.458 ± 0.073 [66]; 0.314 ± 0.028 [21]; 0.425 ± 0.026 [20]
Decay constant of Ca^2+ ^transient (ms)	190	166 ± 12 (0.25 Hz) [66]; 293 ± 19 [20]; 188 ± 14 (0.5 Hz) [19]; 210 (0.5 Hz) [2]
∫I_Ca, L _dt (μM)	1.0	1.5 [22]
Maximum J_REL _(μmol/Lcytosol/ms)	2.2	3 (rat) [72,81]
Diastolic SR Ca^2+ ^content (μM)	92	142 ± 9 [20]; 102 (0.5 Hz) [19]; 64.3 ± 13.0 (0.5 Hz) [73]
Fractional release (%)	28	29 ± 3 [20]
SERCA Ca^2+ ^removal fraction (%)	89	88 [20]; 90.3 (0.5 Hz) [19]; 88 (0.5 Hz) [73]
NCX Ca^2+ ^removal fraction (%)	10	12 [20]; 9.2 (0.5 Hz) [19]; 12 (0.5 Hz) [73]
Slow Ca^2+ ^removal fraction (%)	0.56	0.5 (0.5 Hz) [19]
FDAR (τ_1 Hz_/τ_4 Hz_)	2.36	2.06 ± 0.08 (τ_0.5 Hz_/τ_4 Hz_) [20]; 1.87 ± 0.06 (τ_1 Hz_/τ_5 Hz_) [74]

Simulation results are shown for 1 Hz pacing steady-state. All concentrations are in cytosol volume.

**Figure 3:**
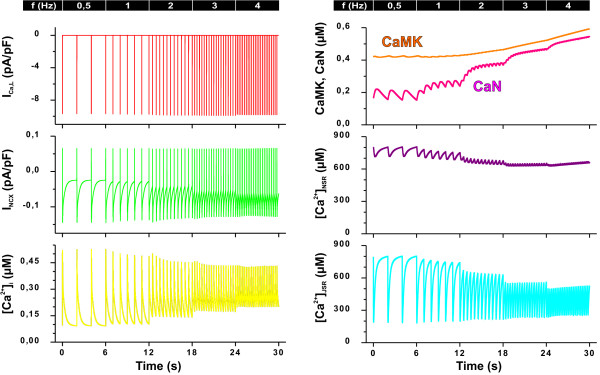
**Examples of outputs (colour coding matches Figure 1) during a dynamic in silico pacing experiment**. The simulation series was started from a steady-state (pacing at 0.5 Hz) and the pacing frequency was increased with six second intervals to 1, 2, 3, and 4 Hz. Left panel shows the L-type calcium current (I_Ca, L_), sodium calcium exchanger current (I_NCX_), and calcium concentration in the cytosol ([Ca^2+^]_i_). Whereas the peak amplitude of I_Ca, L _is hardly changed, the calcium transients become smaller as the pacing frequency increases, because the sarcoplasmic reticulum (SR) Ca^2+ ^stores are reduced. At the same time, the rise of the diastolic [Ca^2+^]_i _causes an increase in the diastolic outward I_NCX_. In the right panel, the concentration of active calcineurin (CaN) and Ca^2+^/calmodulin-dependent protein kinase II (CaMK), as well as the calcium concentration in the network SR (Ca_JSR_) and in the junctional SR (Ca_NSR_), are presented from the same incremental simulation. The CaN curve follows the Ca^2+ ^transients more closely, in fact oscillating clearly at the lowest pacing frequencies, whereas CaMK activity displays a stronger integrative aspect and, accordingly, a much smoother time-course.

To further evaluate the model features, we did a series of simulations aimed to characterize the frequency-dependent changes in the model outputs (Figure 3). Simulations were started from a steady-state (pacing at 0.5 Hz) and the pacing frequency was increased with six second intervals to 1, 2, 3, and 4 Hz. These simulations demonstrate a fundamental feature of the E-C coupling regulation. While the frequency-dependent changes at the level of activity of individual proteins are small, like the change in the L-type calcium current (Figure 3, left upper panel), the sum of these effects is manifested as an order of greater changes in the calcium signals. The calcium transients became smaller as a function of pacing frequency at the same time when the diastolic [Ca^2+^]_i _rose. This shows that even though CaMK increases the activity of the SERCA, it cannot fully compensate for the frequent SR calcium releases with such a short timescale of adaptation (six second intervals). Therefore, both the stored (network sarcoplasmic reticulum; NSR) and releasable (junctional SR; JSR) pools of calcium in the SR are partially depleted, which reduces the SR calcium release while the extra calcium builds up the cytosolic [Ca^2+^]_i_, just like in isolated mouse ventricular myocytes [24,25]. The rise in the diastolic [Ca^2+^]_i_, on the other hand increases the diastolic outward NCX current (Figure 3), which causes a small hyperpolarisation of the resting potential from -79.3 mV at 0.5 Hz to -80.4 mV at 4 Hz.

**Figure 4:**
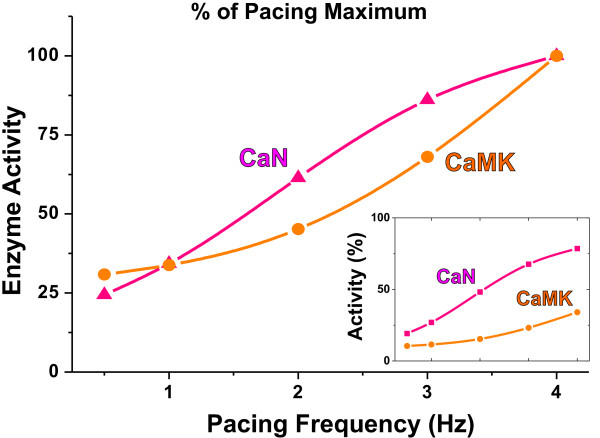
**The simulated concentrations of active CaN and CaMK (colour coding matches Figure 1)**. The enzyme activities at pacing steady-state are shown in relation to the maximum pacing-induced value (at 4 Hz). The divergent pacing dependence of calcineurin (CaN) and Ca^2+^/calmodulin-dependent protein kinase II (CaMK) is demonstrated even more clearly in the inset, which shows the enzyme activities scaled to the maximum calcium-induced value.

As expected, the activity of both CaMK and CaN increases with the pacing frequency from 0.5 Hz to 4 Hz. According to the activation characteristics of CaN, its activity increases the most at pacing frequencies from 0.5 to 2 Hz and starts to saturate at higher frequencies (Figure 3). CaMK, on the other hand, is activated predominantly at frequencies higher than 2 Hz. This feature is identical to the biphasic synaptic plasticity in neurons explained by a model including inhibition of calcium activated CaMK upon co-activated calcineurin [26]. The divergent frequency dependence of CaMK and CaN is highlighted in comparison of the pacing steady-state activities (Figure 4). The difference in the ranges of CaN and CaMK activation is even more evident, when the pacing-dependent enzyme levels are scaled to the theoretical upper limit that can be induced with a maximal calcium stimulus (Figure 4, inset).

### Phospholamban Knockout Model

The elegant cardiac PLB knockout (PLB-KO) mouse model has been used extensively to study the role of the SR in E-C coupling [6,27,28]. One of the rare features of the PLB-KO model among the genetically engineered cardiac mouse models is that the PLB-KO mice do not develop cardiac hypertrophy or failure [6]. PLB-KO can therefore be used in the physiological elucidation of the role of SR calcium uptake modulation via SERCA-PLB interactions as well as the role of SR calcium uptake and release in E-C coupling [12,29-31]. We wanted to test whether our simulations could reproduce the experimental results of this comprehensive mouse model and reveal possible signalling mechanisms that cannot be observed in experiments.

**Figure 5:**
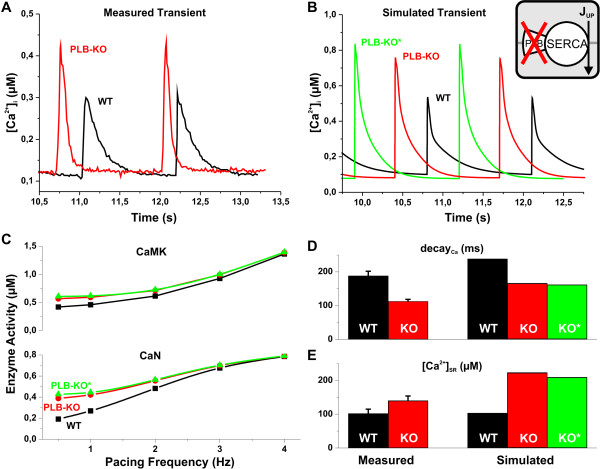
**Comparison of experimental data and simulated results of WT and PLB knockout**. Results of WT, PLB knockout and PLB knockout without compensation are shown with black, red and green symbols, respectively. (A) and (B) Two postrest Ca^2+ ^transients from experimental [32] and simulated data are shown. There is a marked similarity of the shapes of the corresponding Ca^2+ ^transients of the simulated results and the measurements, e.g., the amplitude ratio of PLB-KO vs. WT is 1.6 in both the *in vivo *and the *in silico *experiments. The amplitude of the Ca^2+ ^transient is a further 12% larger in the PLB-KO* test case (knockout with no compensations). (C) Amount of active CaMK and CaN as a function of pacing frequency. Compared to WT the mean activities of CaMK and CaN increase by 35% and 102% for PLB-KO, and by 44% and 122% for PLB-KO* at 0.5 Hz pacing. These deviations from behaviour of the WT myocyte are attenuated when the pacing frequency is increased. (D) Time constants of the [Ca^2+^]_i _transient decay. Measured data [19] is compared to simulated results at 0.5 Hz. The value of *τ *is decreased by 26% and 28% compared to WT for the PLB-KO and PLB-KO* simulations, respectively, and by 40% in the experiments of Li et al. [19]. (E) SR Ca^2+ ^content calculated from measured data [19] and simulated results at 0.5 Hz. The increases of [Ca^2+^]_SR _appears to be exaggerated *in silico*. However, an earlier report by Chu et al. [33] showed an 86% increase of [Ca^2+^]_SR _in the PLB-KO mouse myocytes compared to WT. Thus, the model prediction of the SR calcium content is in good qualitative agreement with the experiments.

Total relief of PLB inhibition of SERCA has a great impact on the Ca^2+ ^dynamics of the myocyte as demonstrated in Figure 5. The simulated results of WT and PLB-KO Ca^2+ ^transients correspond well with the wild-type and knockout measurements of Huser et al. [32]. The comparison of Ca^2+ ^transients in Figure 5A and 5B shows that the amplitude ratio of PLB-KO vs. WT is 1.6 in both the *in vivo *and the *in silico *experiments. It was previously reported that in the PLB-KO mouse, the ventricular cardiomyocytes have a decreased expression level of RyR [33]. We therefore implemented this modification into the model (see Methods for details). With RyR downregulation in the model, the amplitude of the Ca^2+ ^transient is 12% smaller compared to pure phospholamban knock-out due to a reduced amount of calcium release units (Figure 5B). This might indicate that the general mechanism of the compensations is to attenuate the effect of initial modifications, i.e. to reduce the change in myocyte function.

As expected, the increased systolic Ca^2+ ^concentration in PLB-KO causes a substantial rise in the activity of CaMK and CaN (Figure 5C). Compared to WT the mean activities of CaMK and CaN increase by 35% and 102% for PLB-KO, and by 44% and 122% for PLB-KO* (knockout with no compensations) at 0.5 Hz pacing. Upon an increase in the pacing frequency, this difference becomes smaller. Increased amplitude of the Ca^2+ ^transient increases the activity of SERCA both directly (increased systolic [Ca^2+^]_i_) and indirectly (via enzymatic regulation). As a result the decay time constants of the calcium transients decrease by 26% (PLB-KO) and 28% (PLB-KO*) compared to WT (Figure 5D), while the Ca^2+ ^concentration in the SR ([Ca^2+^]_SR_) increases by ~100% in both PLB-KO versions (Figure 5E), which is in line with experimental findings [33].

The increased CaMK activity in the PLB knockout naturally affects the function of LCC as well. Surprisingly, in the simulations the peak value of *I*_*Ca, L *_is actually reduced by 3% in PLB-KO vs. WT simulations for 0.5 Hz pacing. This results from the fact that PLB-KO myocytes have enhanced calcium release due to higher [Ca^2+^]_SR _and increased CaMK phosphorylation of RyR. This ≈ 2-fold increase in J_rel _brings more calcium to the vicinity of the LCCs, thus causing a faster inactivation of the channels. This mechanism has been observed also in experiments [34] and explains elegantly how the complex system controls itself to cope with the changed functional environment.

The overall change in the *in silico *PLB-KO phenotype corresponds very well with *in vivo *results and is rather straightforward, i.e. due to the increased SERCA activity the relaxation of the myocyte is accelerated significantly (Figure 5D). This advantage, however, falls short compared to the physiologically more relevant phenomena. The force-frequency relation (FFR) is reversed: Δ(*F*_*4 Hz *_- *F*_*1 Hz*_) value is +43% and -20% in WT and PLB-KO simulations, respectively. Frequency-dependent acceleration of relaxation (FDAR) is also blunted in the PLB knockout myocytes: the *τ*_0.5 Hz _/*τ*_4 Hz _ratio of calcium transient decay is reduced from 3.5 to 2.3 for WT vs. PLB-KO. However, while the physiological adaptation to increased pacing is impaired, the function of the myocytes is in fact improved; the PLB-KO just recruits the contractile reserve at lower frequencies.

### Transgenic Model of CaMK-Dependent Regulation of L-type Calcium Channel

In order to find out how the function of the cardiac myocyte would change when the CaMK-dependent regulation is switched off, we implemented a transgenic version of the model, in which the modulation of LCC is disrupted either by blocking the phosphorylation (bLCC) or setting the phosphorylation site constitutively active (cLCC); see Methods section for details of implementation. The experimental results indicate that the activation of CaMK promotes longer openings of the L-type calcium channels [14]. At the level of the whole cardiomyocyte, this manifests as slower inactivation and increased peak of the I_Ca, L _current [35]; a phenomenon referred to as "facilitation".

**Figure 6:**
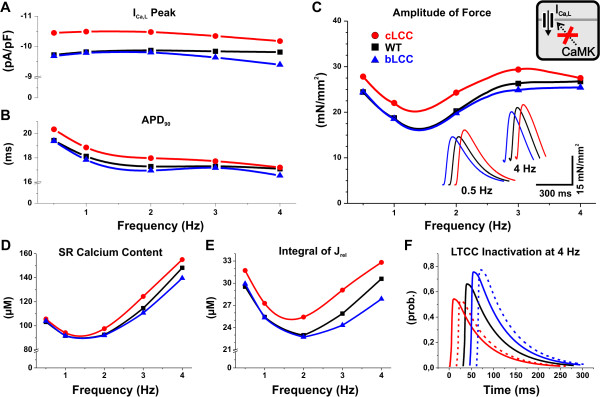
**Comparison of WT and transgenic LCC models**. The results of TG models with constitutively active CaMK-dependent phosphorylation (cLCC) and blocked phosphorylation (bLCC) of LCC are depicted with red circles and blue triangles, respectively. These are compared to simulation results of wild-type model (black squares). (A) Peak value of L-type calcium current (I_Ca, L_) as a function of pacing frequency. The I_Ca, L _peak amplitude of bLCC model deviates rather little from the WT (-4.3% at 4 Hz). In the cLCC model, the peak value of I_Ca, L _is larger than in the WT (+7.5% at 0.5 Hz pacing), but the deviation decreases as pacing frequency increases. (B) Action potential duration (90% recovery, APD_90_) presented as a function of pacing frequency. Compared to WT, the APD_90 _is virtually unchanged in both TG models. (C) Contraction force amplitude as a function of pacing frequency. The difference in the force amplitude is also very small: -4.9% for bLCC (at 4 Hz) and +14% for cLCC (at 0.5 Hz). Inset shows twitch force of the WT and TG model at 0.5 Hz and 4 Hz pacing frequencies. (D) and (E) SR Ca^2+ ^content ([Ca^2+^]_SR_) and the integral of Ca^2+ ^release from the SR (J_rel_) during one AP plotted as a function of pacing frequency. There is very little change in the [Ca^2+^]_SR _at low pacing frequencies. The maximum deviation from WT is seen at 4 Hz pacing: -6% and +5% for bLCC and cLCC, respectively. The integral of J_rel _changes slightly more -9% and +7% for bLCC and cLCC, respectively. (F) Demonstration of the autoregulation involving I_Ca, L _and J_rel_. Dashed lines present acute transgenic situation, in which the LCC inactivation is calculated from the WT parameter values. Note that the time axis of the traces has been shifted for easier comparison.

According to simulations, the disrupted CaMK-dependent modulation of LCC changes the frequency-dependence of I_Ca, L _(Figure 6A). The maximum deviation of the amplitude of I_Ca, L _from WT is -4.3% (at 4 Hz) and +7.5% (at 0.5 Hz) for bLCC and cLCC, respectively. The contractile force, which can be considered as a characteristic output of the end-point phenotype, is affected only to a very small extent (Figure 6C). The inset of Figure 6C further indicates that the dynamics of the force are not drastically affected. That is, the main changing variable is the amplitude of the force, whereas, the baseline tension, time-to-peak-force and relaxation time remain practically constant compared to WT. As one could expect for a short action potential animal such as the mouse, the AP duration (at 90% repolarization) is virtually unaltered between the transgenic and wild-type models (Figure 6B).

The increased (in cLCC) and decreased (in bLCC) I_Ca, L _should lead to accumulation and depletion of intracellular calcium, respectively. In the simulation results, this is seen as altered SR calcium content (Figure 6D) and consequent changes in the integral of J_rel _(Figure 6E). These two parameters behave differently in function of the pacing frequency. While the deviation of bLCC from WT is parallel to the changes of I_Ca, L _(Figure 6E), the difference between cLCC and WT is more pronounced in the integral of J_rel_. This is due to the fact that calcium release from the SR is affected not only by the increased SR calcium content but also by the enhanced trigger, i.e. I_Ca, L_.

Intuitively, the changes in the calcium current should perhaps lead to more significant changes in the E-C coupling, since LCC is one of the main components of cellular calcium transport machinery. Furthermore, the strong coupling of [Ca^2+^]_i _and CaMK activation, decreased in bLCC and increased in cLCC, should work to enhance those alterations due to consequent changes in the phosphorylation of the other CaMK targets RyR and SERCA. This scenario is, however, affected by another regulatory loop. In the bLCC case, decreased I_Ca, L _leads to decreased J_rel_, which reduces the calcium-dependent inactivation of LCC; a chain of events that affects the intracellular calcium dynamics in the opposite direction in cLCC. Thus, in both cases this autoregulation would tend to reduce the original effect of altered I_Ca, L_. To demonstrate this, we calculated what the inactivation of LCC would be in the transgenic models if all the other parameters were identical to WT (Figure 6F; dashed lines); see Methods section for details of this acute TG. This shows that with the feedback systems present, the autoregulation mechanisms bring the system closer to WT behaviour. Thus, a change in one physiological regulatory event, in this case the disrupted modulation of the LCC, cannot easily induce drastic changes to the overall function of the myocyte.

### CaMK Overexpression Model

CaMK has a substantial role in the E-C coupling of both the normal and the failing heart [36-39]. Cardiac overexpression of the cytosolic isoform of CaMK results in cardiac hypertrophy and a unique phenotype of the myocytes [20]. The phenotype resulted from the initial modification (3× increase in the CaMK expression), but also from a variety of significant changes in the expressions of E-C coupling proteins, like SERCA, RyR and NCX [20]; see Methods for details. To elucidate the relative contribution of these two mechanisms, i.e., the CaMK overexpression and the compensatory changes, we implemented two versions of the *in silico *model. The first one (CaMK3X) simulates the experimentally observed phenotype and the second one (CaMK3X*) a theoretical situation, where there are no compensations at the level of gene expression (implementation scheme can be found in Methods).

**Figure 7:**
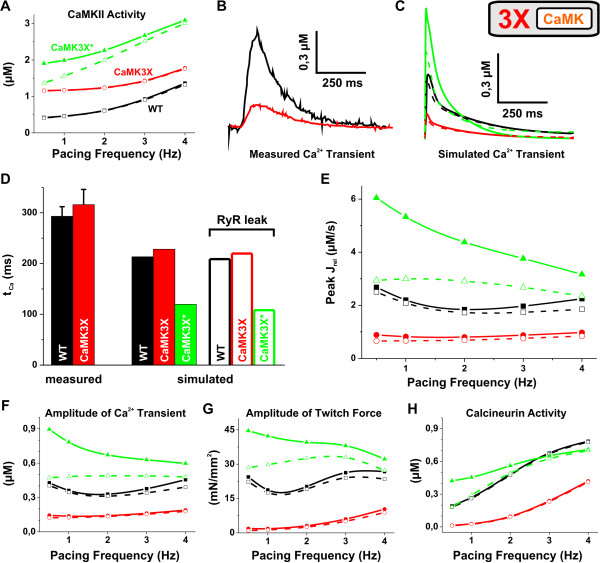
**Comparison of experimental data and simulated results of WT and CaMK**. Black (squares), red (circles) and green (triangles) symbols indicate WT, CaMK overexpression (CaMK3X) and CaMK overexpression with no compensations (CaMK3X*), respectively. The corresponding test cases without and with CaMK induced SR leak are depicted with closed symbols & continuous lines and open symbols & dashed lines. (A) CaMK activity as a function of pacing frequency. The pacing-induced (0.5 to 4 Hz) increase of CaMK activity is 35% smaller for CaMK3X vs. WT. (B) and (C) [Ca^r+^]_i _transient at 1 Hz. Compared to WT, the amplitude of the measured [20] and the simulated transient changes by -77% and -62% due to CaMK overexpression, respectively, and CaMK3X* by +117%. (D) Time constants of the [Ca^2+^]_i _transient decay. At 1 Hz, the combination of CaMK overexpression and compensational changes increases the time constant of measured [20] and simulated Ca^2+ ^transient decay by 7.8% and 14%, respectively. Whereas, the mere overexpression (CaMK3X*) decreases *τ*_Ca _by 41% compared to WT. (E) Peak value of SR calcium release as function of pacing frequency. WT and CaMK3X were not affected to a large extent by the leakiness of RyR. Whereas, in the CaMK3X* test case, the behaviour changes strikingly. (F) and (G) The amplitudes of Ca^2+ ^transient and contractile force as a function of pacing frequency. A "leaky" RyR tends to normalize the functionality of the CaMK3X* test case, but has little effect in WT and CaMK3X. (H) Activity of CaN as a function of pacing frequency. At 1 Hz pacing, CaN activity for CaMK3X is reduced to 10% of the WT value and increased by 69% for CaMK3X*. Increased pacing is unable restore CaN of CaMK3X to the WT level, whereas the deviation of CaMK3X* from WT is reversed by faster pacing. Interestingly, the RyR leakiness brings the CaN-frequency curve of CaMK3X* closer to WT.

When the total amount of CaMK was increased 3× corresponding to the level of the CaMK in the CaMK-TG cardiomyocytes, the amount of active CaMK was changed dramatically at any given pacing frequency in the model (Figure 6A). At 1 Hz pacing, CaMK activities were 2.5 and 4.3 times higher than WT in the CaMK3X and the CaMK3X*, respectively. When CaMK activity is forced to such a high level, increased phosphorylation of CaMK targets should increase the calcium influx (via LCC), and increase SR calcium uptake (via PLB and SERCA) and release (via RyR), which together should increase the calcium signals dramatically. Therefore the reported CaMK-TG mouse cardiomyocyte phenotype seems counterintuitive, because TG myocytes have dramatically decreased Ca^2+ ^transient amplitudes (Figure 7B). Contrary to this, the model predicts that CaMK overexpression alone (CaMK3X*) increases the calcium transient amplitude by 117%, and contraction force by 126% (Figure 7C), while the calcium transient decay (τ_Ca_) decreases simultaneously by 41% compared to WT at 1 Hz (Figure 7D).

At the level of cytosolic calcium signals and contraction, these changes are qualitatively almost identical to those induced by deletion of PLB (Figure 5B). However, while the compensations in PLB-KO myocytes are minute and the phenotype is mainly due to ablation of PLB, the CaMK-TG phenotype seems to be induced mostly by secondary changes in gene expression. That is, when the documented changes in the protein levels of CaMK-TG myocytes were implemented into the model, the amplitude of the simulated calcium transients decreased by 62% and the contraction force by 90% at 1 Hz pacing compared to the WT model. Quite naturally the smaller calcium signals also affect the amount of calcium activated CaMK, which is reduced by 41% compared to the CaMK3X*. Again this demonstrates a general feature of biological feedback loops, where secondary modifications tend to reduce the strength of the initial stimulus. Despite the dramatically increased CaMK phosphorylation, which promotes activation of RyR, the amount of calcium released from the SR is significantly smaller (55% decrease at 1 Hz pacing) in the CaMK3X (Figure 7E). Thus, the reduction in the [Ca^2+^]_SR _prevents an increase in calcium release (J_rel_). This conclusion is supported by the fact that the fractional release is reduced by 40% from 0.28 to 0.17 *in silico*.

In the model with CaMK overexpression and the reported changes in the protein levels, SR calcium content is reduced by 25% (data not shown). However, it was reported earlier that *in vivo *the reduction was 44% [20]. To explain the prominent reduction of the SR calcium content, it was reported that the diastolic Ca^2+ ^leak was increased 4-fold in the TG vs. WT mice [20]. To account for this mechanism, we updated the model with a putative CaMK-dependent RyR leak (see Methods section for details), which produced a ≈ 3-fold increase in the resting Ca^2+ ^leak for CaMK3X vs. WT (data not shown). When simulated together with the CaMK3X model version, the RyR leak did not induce a significant effect on the already depressed E-C coupling. We found that the SR Ca^2+ ^content was reduced by 17% in the leaky CaMK3X compared to the non-leaky version, but the Ca^2+ ^transient amplitude and contraction force were decreased only slightly (Figure 7F and 7G). According to the simulations, the WT *in silico *myocyte was not affected to a large extent by the leakiness either.

When leaky RyRs were simulated together with the acute effect of CaMK overexpression (CaMK3X*), calcium signalling was drastically changed. As expected, upon an increase in the RyR leak, the SR calcium content was decreased by 38% (at 1 Hz), leading to reduced SR calcium release and subsequently smaller calcium transients (Figure 7C). However, it is quite unexpected that according to simulations the RyR leak might have some beneficial effects on the function of cardiomyocytes facing an acute increase in CaMK activity. While limiting the increase in the calcium transient amplitude, the RyR leak enhances the calcium transient decay (Figure 7D) and, more importantly, restores the positive trend of the force-frequency-relationship (Figure 7F and 7G). The physiological adaptation to an increase in the contraction rate is thus re-established. This data is qualitatively in line with the results from a robust acute overexpression of CaMK in rabbit cardiomyocytes [40]. It was reported that despite a 5- to 6-fold increase in the amount of active CaMK, the contractility was unaltered because the CaMK-dependent RyR phosphorylation was increased and consequently SR Ca^2+ ^leak was greatly augmented [40]. Our modelling implies that in the acute overexpression state the CaMK-dependent the RyR leak might work as a compensatory mechanism to restore some features of E-C coupling by limiting the effects of overly enhanced CaMK activity on the calcium signals.

CaMK overexpression simulations also give predictions of how the altered calcium signalling affects the activity of other calcium-activated enzymes. For example, calcineurin activity, which is a master regulator of the expression of cardiac genes [41] and can promote cardiac hypertrophy and failure [8], is indirectly modulated by CaMK activity. The augmented calcium signals in CaMK3X* simulations increase the CaN activity by 69% and conversely the blunted calcium signals in CaMK3X simulations reduce the CaN activity by 90% at 1 Hz pacing compared to WT. Interestingly, when the normal pacing induced calcium changes are impaired in the CaMK3X model, the pacing induced increase in the CaN activity (from 0.5 Hz to 4 Hz) is decreased by 32% (Figure 7H). Consequently, the CaN activity is lower even at high pacing frequencies (47% reduction at 4 Hz) compared to WT. Another interesting observation is that the putative CaMK-induced RyR leak tends to normalize the CaN activity in the CaMK3X* test case, i.e. the pacing induced changes are more similar to WT. This is again an example of a mechanism that could help a complex biological system maintain its physiological function in spite of genetic interventions.

## Discussion

Our results highlight the intrinsic complexity of cardiomyocyte E-C-coupling, which originates from the interdependencies of regulatory mechanisms involving calcium and the components regulating the membrane excitability. Facing this complexity, experimental observations, even clever and ingenious ones, may not be enough to reveal the causal connection of the cellular signalling pathways, where networks operating in parallel form feedback loops that control the dynamic physiological features of the cells. These complex networks give some fundamental and unanticipated properties to the cell function. For example, they make the system more robust, i.e., tolerant to change in the single part of a signalling network [42]. Even more challenging task is faced when the networks are interfered like in studies of genetically engineered models. When some cellular function is disrupted by genetic intervention, the resulting phenotype is not necessarily exactly what was expected. Instead, homeostatic regulatory mechanisms in cells tend to minimize the effects of the mutation on the phenotype.

The presented model is the first mathematical model of mouse ventricular myocyte E-C coupling that also includes the regulation of the calcium transport machinery through the activation of CaMK. The model has properties common to complex biological systems, which explain the basic features of cardiomyocyte function: 1) pacing-induced changes in calcium dynamics via CaMK-dependent regulation, as well as 2) robustness against experimental interventions (caffeine-induced calcium release) and genetic modifications (PLBKO and disruption of CaMK-dependent modulation of LCC). The simulations with the model give fundamental information on interrelations between different signalling pathways in cardiomyocyte E-C coupling and clarify the role of parallel feedback loops in maintaining the operation of cardiomyocyte E-C coupling. We showed that through the feedback loops cardiomyocytes are surprisingly resistant to alterations in the activity of single endpoint components in the signalling networks, whereas interventions disrupting the feedback loops will compromise the function. According to our simulations, these inherited properties of cardiomyocyte E-C coupling are likely to define the impact of genetic manipulations on the end-phenotype of the cardiomyocytes.

### Limitations of the Study

The fundamental challenge in developing an E-C coupling model is the rather large variability of experimental data. Therefore, it would be an irrelevant and futile effort to try to fit the behaviour of the model perfectly to one single set of *in vivo *data. Instead, it is more essential that the outputs of the model agree qualitatively with the majority of the measured results, as is the case with the three fundamental outputs (AP, Ca^2+ ^transient and force) in our model. Furthermore, the model was also able to simulate faithfully such multivariable cellular phenomena as the force-frequency relationships and the caffeine pulse experiments. This kind of high-level validation is crucial for a model that is comprised of multitude of submodels, because for most of the model parameters there is no corresponding measurement data, to which the parameter values could be directly fitted; a second fundamental challenge in this modelling field.

The isoform-specific differences of the CaMK reaction chain in the neuronal vs. cardiac tissue warrant further studies. However, a fair assumption, one that we have made, is that these isoforms of the kinase behave in a rather similar manner, since they share 89-93% sequence similarity in their catalytic and autoregulatory domains [43]. This issue was recently studied by Chiba et al. with a mathematical model that included the CaMK-CaM interaction dynamics specific to neurons and cardiomyocytes [44]. Our approach to describe this isoform divergence is, albeit non-mechanistic, a coherent one (see Methods section for details). The results of Chiba et al. [44] and Saucerman et al. [45] also underline the significant role of PP1 activity in CaMK dynamics. In addition to PP1, the enzyme reaction network of Bhalla and Iyengar [46] includes also the other two main phosphatases, CaN and PP2A, found in cardiac myocytes. Thus, it is a comprehensive description of enzyme kinetics with notable application potential in future studies as well.

Despite the limitations mentioned above, our model reproduces faithfully the main features E-C coupling of mouse ventricular myocytes. Simulation results also suggest that, despite or because of the complexity of the model, it behaves robustly enough to be a suitable and valid platform to study the physiological and pathophysiological phenomena in mouse cardiac myocytes. Especially interesting issue that can be targeted with our model is the role of CaMK in various heart failure conditions.

### Physiological Role of CaMK in Cardiomyocytes

CaMK is a multifunctional holoenzyme that has modulates E-C coupling by phosphorylating SERCA, PLB, RyR and LCC [14,40,47,48]. CaMK goes through a process of autophosphorylation, i.e. the phosphorylated state continues even after the Ca^2+^-CaM complex has been removed, which leads to prolonged activation of CaMK [49]. It has been shown that the enzyme can decode the frequency and the amplitude of Ca^2+ ^spikes into distinct levels of kinase activity [16]. With this unique combination of features CaMK mediates the beating frequency-dependent regulation in cardiac myocytes and establishes a feedback signalling network that controls cardiomyocyte Ca^2+ ^signals and excitability. Apart from this physiological function, CaMK has been verified as a proarrhythmic signalling molecule and may therefore offer novel solutions for antiarrhythmic therapy [35,50]. In this study, we focused on the CaMK-dependent regulation of E-C coupling. The presented mathematical model was validated both in WT and transgenic test cases. Thus, it forms a good base for further studies of the role of CaMK, and CaN, e.g. in arrhythmia and excitation-transcription coupling.

### Phospholamban Knockout Model

The phospholamban knockout model is one of the few reported mouse models, where genetic manipulation produces enhancement of the cardiac function instead of pathological developments and failure. In theory, the absence of PLB-dependent inhibition of SERCA should increase the SR calcium uptake, which would result in faster decay of calcium transients, elevated [Ca^2+^]_SR _and a subsequent increases in the amplitudes of the calcium transients. The analysis of the PLB-KO mice showed that all of the expected changes were present in the PLB-KO cardiac myocytes and that the PLB ablation had induced only very mild phenotypical compensations [19,33]. In our model, the experimental results from the PLB-KO mouse were mostly reproduced by ablation of the PLB, and the reported compensatory reduction of the RyR expression did not significantly change the E-C coupling.

The enhanced systolic [Ca^2+^]_i _also activates both of the calcium-dependent enzymes in the model. Because CaMK is predominantly activated at relatively high frequencies (Figure 4), increase of the amplitude of calcium transient at low frequencies elevates CaMK activity only modestly (35% at 0.5 Hz) compared to calcineurin activity, which is doubled at low frequency (102% increase at 0.5 Hz pacing). Since calcineurin regulates cardiac gene expression [41] and promotes cardiac hypertrophy and even failure [8], enhanced calcineurin activity should have a great impact on the cardiac phenotype. However, according to the simulations presented here, at higher frequencies both the CaMK and CaN activities of PLB-KO myocytes are normalized. At 4 Hz, the enzyme activities of PLB-KO and WT myocytes are identical, suggesting that at normal mouse heart rates (5-10 Hz) CaMK and CaN are not changed in PLB-KO hearts.

From the physiological point of view, ablation of PLB causes the myocyte to recruit more calcium-activated force than would be required at low pacing frequencies. This change alone increases the energy consumption of the myocyte, since the contractile element and SERCA are the main energy consuming components of the system. Therefore it is a surprise that the cardiac phenotype of the PLB-KO mouse is so close to the WT and without pathological changes; a finding that has been shown to endure even in the long-term [51].

### Transgenic Model of CaMK-Dependent Regulation of L-type Calcium Channel

Considering the fundamental role that I_Ca, L _has in E-C coupling as the initiator of the CICR process, the enzymatic regulation of LCCs is an extremely interesting subject for in silico studies. The CaMK-dependent facilitation of I_Ca, L _is considered to be an important part of the physiological regulation of E-C coupling (for review see [52]) but it may also be involved in arrhythmias [36].

Despite the crucial role of I_Ca, L_, its CaMK-dependent regulation appears to be less essential for the regulation of E-C coupling in mouse ventricular myocytes. In both cases, bLCC and cLCC, with disrupted CaMK-dependent modulation the autoregulatory link between I_Ca, L _and J_rel _tends to reduce the original effect of altered I_Ca, L_, thus bringing the TG system closer to WT behaviour. This is a good example of how difficult it is to combine a holistic point of view based on the data from experimental findings. For example, it has been reported that the super-maximal change of I_Ca, L _induced by the 5-6 fold overexpression of CaMK is 22% of the calcium current [40]. If we accept that this effect is significantly higher than the modulation induced by maximally activated endogenous CaMK, which would occur at the highest cardiomyocyte beating rates (mouse ~10 Hz), then the CaMK-induced modulation of I_Ca, L _at pacing rates applicable to isolated mouse cardiomyocytes (0.5-2 Hz) would be minute. In addition, in experiments all other normal cellular feedback systems controlling I_Ca, L _will be excluded, which further biases evaluation of the potential impact of the CaMK modulation on the I_Ca, L _and especially on the function of the whole cell. This was well demonstrated by the model, where the large CaMK facilitation could be expected to produce a large modification of E-C coupling, but *in silico *the effect turned out to be rather small.

### CaMK Overexpression Model

To study the possible role of CaMK activity in the development of hypertrophy and failure, a mouse model overexpressing cytosolic CaMK was generated by Maier et al. [20]. This genetic intervention results in unique changes in cardiomyocyte calcium signalling, cardiac hypertrophy and dilated heart failure [20], suggesting that chronic CaMK overexpression triggers drastic alteration in cardiomyocyte gene expression.

In our model, an increase in the amount of CaMK has dramatic effects on myocyte function through an increase in the CaMK activity and subsequent activation of CaMK targets. This leads to greatly augmented calcium release and uptake resulting in enhanced calcium signals. These are quite the opposite to the reported phenotype of the CaMK overexpression mouse, suggesting that the compensatory changes in the gene expression have more impact on the CaMK-TG phenotype than the CaMK-dependent regulative mechanisms *per se*. Supporting this, when the protein expression changes of the CaMK-TG myocytes reported in [20] were incorporated into the model, our simulations produced blunted calcium signals, impaired force-frequency and calcium-frequency relations, just like in the experiments with CaMK-TG myocytes [20].

In order to understand the sequence of events leading to the endpoint phenotype, the acute effect of the genetic modification should be explored. Acute effects of CaMK were studied recently by adenoviral overexpression (5- to 6-fold) of CaMK in isolated rabbit cardiomyocytes [40]. Surprisingly, CaMK overexpressing myocytes had normal calcium transient amplitudes and reduced SR calcium content [40]. To explain this it was suggested that the CaMK-induced RyR phosphorylation would increase the SR calcium leak, which in turn would reduce SR calcium load despite the CaMK-dependent increase in the SR calcium uptake [20,40]. In our simulations, the CaMK-induced RyR leak during CaMK overexpression (CaMK3X* test case) reduced the SR calcium content and release, in line with the experimental findings [20,40]. This leads to secondary effects: 1) due to smaller calcium transients compared to CaMK overexpression without the RyR leak, the amount of active CaMK and CaN are reduced and 2) the negative trend of the force-frequency relation is almost reversed. Thus, it would seem that in the acute phase of the overexpression state the CaMK-dependent RyR leak might be beneficial for the myocyte function, since it acts to limit the overly enhanced calcium signals. Due to the CaMK-induced RyR calcium leak, the initial change of the CaMK overexpression in the calcium signals is modest.

Why then does CaMK overexpression induce such extensive compensations at the level of transcription? CaMK overexpression differs fundamentally from PLB-KO in that it switches off the whole physiological feedback system between the cytosolic calcium signals and the CaMK. Therefore, it is likely that these sorts of manipulations, which destroy the ability of the cell to adapt to the functional demands, will trigger profound transcriptional compensations.

## Conclusion

Mathematical modelling can be exploited as an integrative tool to dissect the underlying processes of cellular function in both physiological and pathophysiological situations. The potential of *in silico *studies lays in the ability to simultaneously observe multiple variables and to estimate such outputs that cannot be measured in the experiments. Our simulations show that modelling enables conclusions about the causalities between myocyte signalling cascades, which would be challenging to reach solely by interpretation and analysis of *in vivo *measurements. Thus, it is possible to gain more information of the 'autoregulatory' phenomenon in the cardiac myocyte. In the present study, we have demonstrated this for normal and three transgenic cell types.

## Methods

We base our model of ventricular myocytes of adult mouse on three existing model components: 1) a model of cardiomyocyte electrophysiology by Bondarenko et al. [53], 2) a description of the contractile element by Cortassa et al. [54] and 3) a biochemical scheme of CaMK activation by Bhalla and Iyengar [46]. A schematic drawing of the developed model is presented in Figure 1.

The model parameters were adjusted, whenever possible, according to experimental data (as specified in Tables 1, 2, 3 &4[55-75] or in the following sub-chapters) that was either retrieved directly from literature as numerical values or digitized from visualised data. The parameter values were estimated by direct adapting from literature (e.g., the K_m _value of SERCA) or indirect fitting (e.g., for maximum pump rate of SERCA based on the decay of Ca^2+ ^transient); see Table 3 for a complete list of modified parameters. In either case, the fit was considered satisfactory when the values and the curves of the fits were within the standard error of the mean of the experimental values; see Table 2. In line with most common experimental settings, the model is nominally adjusted for a room temperature of 25°C.

**Table 3:** Modified parameter values of the previously published model components

Symbol	Description	Criteria for Modification	Value
*G*_*Na*_	Maximum conductance of fast Na^+ ^current (mS/μF)	Increasing dV/dt_max _of V_m_	14.5
	Maximum Ca^2+ ^transport rate of PMCA (pA/pF)	Adjusting Ca^2+ ^circulation fraction	0.05
*G*_*Cab*_	Conductance of background Ca^2+ ^current (mS/μF)	Adjusting SR calcium content	3.4 × 10^-4^
	Maximum conductance of NKA (pA/pF)	Matching to recent data of ref. [67]	1.4
*K*_*m, Nai*_	Half maximal [Na^+^]_i _sensitive activation (mM)	(same as above)	18.6
*n*_*NKA*_	Exponent of NKA Hill equation	(same as above)	3.2
	Ca^2+ ^on-rate for troponin high-affinity sites (μM^-1^ms^-1^)	Adapted from ref. [81]	2.37 × 10^-3^
	Ca^2+ ^off-rate for troponin high-affinity sites (ms^-1^)	(same as above)	3.2 × 10^-5^
	Ca^2+ ^on-rate for troponin low-affinity sites (μM^-1^ms^-1^)	Lowering of the baseline tension according to data of [21,22]	0.07
*SL*	Sarcomere length (μm)	Adapted from ref. [22]	2.0
*f*_*φ*_	Scaling factor for the speed of contraction generation'(see eq. A111 in ref.)	Matching the force transient to data of refs. [21,22]	2.3
	Scaling factor for the half maximal transition rate of tropomyosin from permissive to nonpermissive (see eq. A112 and A113 in ref.)	(same as above)	2.8

**Table 4:** Initial conditions for the differential parameters

Parameter	Description	Value (μM)	Refs.
CaM	Calmodulin	6	[75]
CaMK	Basal activity of Ca^2+^/Calmodulin-dependent Kinase	10*	[17]
total_CaMK	Total amount of Ca^2+^/Calmodulin-dependent Kinase (buffered)	10*	[17]
CaNAB_Ca2	Calcineurin with two Ca^2+^-sites occupied	1	[46]
I1	Unphosphorylated form of Protein Phosphatase Inhibitor-1	1.8	[46]
I1_x	Phosphorylated form of Protein Phosphatase Inhibitor-1	0.001	[46]
PP1_active	Phosphorylated form of Protein Phosphatase 1	1.8	[46]
tot_PP1	Total concentration of Protein Phosphatase 2A	1.8	[17]
PP2A	Phosphorylated form of Protein Phosphatase 2A	0.12	[46]
PKA_active	Phosphorylated form of Protein Kinase A (buffered)	0.014735	[17]
---	Parameters of the action potential model.	---	[53]
---	Parameters of the contraction model.	---	[54]

* A simplified approach to represent the 10 subunits, when CaMK concentration is 1 μM.

### Model of Cardiomyocyte Electrophysiology

**Figure 8:**
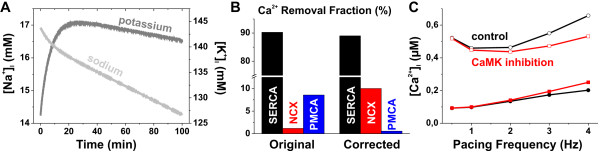
**Stability of the model and characteristics of the CaMK-dependent calcium dynamics**. (A) Intracellular Na^+ ^and K^+ ^concentrations drift during a normal pacing stimulation at 1 Hz with the original Bondarenko model [53] and the model actually never stabilizes to physiological steady-state. Table 1 shows the corresponding values obtained with the developed model. (B) In the original Bondarenko model the removal fractions of NCX and PMCA were distorted reversely; see also Table 2. (C) Inclusion of the CaMK-dependent regulation of the calcium transport mechanisms enhances the positive dependence of systolic calcium (open symbols) on the pacing frequency. It also limits the excess rise of diastolic calcium (closed symbols) with high pacing frequencies, in line with the experimental findings (see Figure 2B).

Due to the employment of a non-conservative stimulus current, the original mouse cardiomyocyte model by Bondarenko et al. [53] had serious stability issues that manifested as a chronic fluctuation of intracellular [K^+^] and [Na^+^]; see Figure 8A. To increase the pacing steady state stability of the model, we implemented a conservative stimulation scheme, in which the stimulus current is carried by K^+ ^ions as suggested by Hund et al. [76]. This approach assured that the simulation results could indeed be obtained at a pacing steady state.

We have also made some modifications to the features and parameters of the Bondarenko model to obtain a better fit to the physiological data of mouse myocytes. A comprehensive improvement was the adjustment of calcium removal fractions of SERCA, NCX and plasma membrane Ca^2+ ^ATPase (PMCA). The original set of parameters caused an imbalance between NCX and PMCA; see Figure 8B and Table 2. The modifications are described in detail in the following subchapters; see also Table 3.

### CaMK Reaction Scheme

The CaMK part of the model is based on a previously published reaction scheme [46]. Information on the detailed structure of the reaction pathways, parameter values and computer source codes are available in the DOCQS Database [77]. The model of the CaMK reaction chain was originally designed for neurons, but its applicability for cardiac myocytes has already been documented in our previous studies [41]. Original values were used for reaction rate constants, but initial concentrations of some variables have been adjusted to the physiology of cardiac myocytes (see Table 4 for details). Essentially, the CaMK reaction scheme published by Bhalla et al. [46] is a highly detailed mathematical description (45 differential equations) based on extensive biochemical studies, thus, it offers novel possibilities for studying the regulation of E-C coupling in a way not possible for example with the simplified (one differential equation) scheme introduced by Hund et al. [78]. A good example of this potential is the overexpression of CaMK that is investigated in this paper.

In Figure 1, the CaMK submodel is presented as a simplified control diagram. A rise in the [Ca^2+^]_i _level increases Ca^2+ ^binding to CaM, which in turn phosphorylates more CaMK and CaN. Phosphorylation of the latter induces phosphorylation of protein phosphatase 1 (PP1). The autophosphorylation of CaMK is presented as a positive feedback loop and PP1 inhibition as negative feedback. The input for the enzyme reactions is [Ca^2+^]_i _upscaled by a factor of two. This is a non-mechanistic approach; however, it emulates the higher sensitivity of cardiac vs. neuronal CaMK isoform to [Ca^2+^], which was studied in detail by Chiba et al. in their recent work [44]. They reported that the calcium concentration value of half-maximal activation of CaMK autophosphorylation is ~2.5 μM and ~1.2 μM for the neuronal and cardiac isoform, respectively. Thus, the scaling factor of two matches well to the findings. It is also in line with the assumption that CaMK is located in such calcium signalling sub-domains, in which calcium concentration reaches, on average, higher peak values than in the bulk cytosolic compartment.

The regulatory effect that CaMK has on SERCA, PLB, LCC and RyR is calculated from the level of active CaMK in the cytosol with the following equation

(2)

where the half maximal CaMK activity, *K*_*m, CaMK *_= 1.2 μM and *n *= 3. We tested a variety of values for these two parameters and the best fit to experimental data of the frequency-dependence of the intracellular calcium dynamics (see Figure 2B), and FDAR (see Table 2) was obtained with these values.

The effect of CaMK-dependent regulation of calcium transport mechanisms is demonstrated in Figure 8C. The two sets of traces refer to simulations run with either a normal model scheme (wild-type; black) or a scheme, in which the CaMK activity has been "clamped" to the quiescent level (CaMK inhibition; red). As the comparison with Figure 2B demonstrates, inclusion of the CaMK-dependent regulation of the calcium transport mechanisms is essential for the positive dependence of calcium transient amplitude on the pacing frequency. The regulation also limits the excess rise of diastolic calcium.

### Sarcoplasmic Reticulum Ca^2+ ^ATPase

The Ca^2+ ^uptake to SR, i.e. the flux *J*_*up *_driven by SERCA, is described with an equation that was originally formulated by Jafri et al. [79]. In our model, we modified that equation by using the same principles that Hund et al. used in their model of canine myocytes [78]. Parameters defining the direct CaMK modulation of SERCA as well as the indirect modulation via PLB are included in the equation

(3)

The maximal relative change in the uptake rate that CaMK can induce, , was set to 70% according to the experimental findings of Toyofuku et al. [47]. Whether CaMK increases Ca^2+ ^uptake by SERCA or not has been a controversial issue according experimental findings. Both an increase [47] and a null-effect [80] have been reported. However, more recent *in vivo *experimental results support the positive effect of CaMK. Phosphorylation of PLB by CaMK relieves its inhibitory effect on SERCA. After testing a variety of values, we set the maximal change in the *K*_*m, PLB *_value to 0.17 μM, which is consistent with the experimental results of Odermatt et al. [80].

In addition to implementation of the CaMK dependence, we also decreased the value of *V*_*max *_to 0.16 μM/ms. This was done to increase the decay time constant of the Ca^2+ ^transient, to correct the Ca^2+ ^recirculation fractions of SERCA, NCX and PMCA, and to lower the SR Ca^2+ ^content to a more physiological level (Table 2). Based on a wide-ranging iteration of the apparent *K*_*m *_value of SERCA, we decreased *K*_*m, up *_to 0.33 μM and the exponent *n *to 1.8. These values gave the best overall fit to experimental data available on SERCA and they also corresponded well to the values suggested for all species [81]. The *K*_*m, up *_value is somewhat higher than the values 0.23 ± 0.04 μM and 0.20 ± 0.04 μM measured from mouse myocytes by Frank et al. [29], but this difference is reduced by CaMK regulation.

### L-Type Calcium Channel

Dzhura et al. have shown that in single channel measurements CaMK promotes longer openings of LCC, i.e. gating mode 2 [14]. At whole cell level, this is seen as slowed inactivation [82]. Thus, in a deterministic modelling setting the latter mechanisms is a feasible way to represent the CaMK-dependent modulation. Based on experimental results we made the γ parameter in the Markov model (see Figure 4 in ref. [53]) dependent on CaMK regulation according to equation (4). Parameter *K*_*pc, max *_defines the maximum time constant for Ca^2+ ^-induced inactivation and *K*_*pc, half *_the corresponding half-saturation level. The CaMK modulation of parameter *γ *thus slows down the inactivation of LCC.

(4)

Some experiments indicate that *I*_*Ca, L *_can be increased by CaMK modulation as much as 40% to 50% [83,84]. However, in light of the most recent experimental results, the modulatory effect may be smaller. Gao et al. reported that the widely used CaMK inhibitor KN-93 has a CaMK-independent inhibitory effect on LCC [85]. They found that the inhibitory effect of a specific inhibitory peptide was less than one third of the inhibitory effect that KN-93 had on LCC [85]. Peptide inhibition studies done with rabbit cardiac myocytes acutely overexpressing CaMK also indicate a lesser modulatory increase of about 22% [40]. Based on these experimental data, we set the modulation of parameter *γ *so that the maximal CaMK-dependent increase in *I*_*Ca, L *_amplitude is ~10%.

### Ryanodine Receptor Calcium Channel

Until recently, the effect of CaMK regulation on RyR (i.e. Ca^2+ ^release from the SR) has been a controversial issue. While some studies have shown that CaMK decreases *J*_*rel *_[86], other studies have reported the opposite result [20]. More recent experimental results [40] and modelling analysis [62], however, strongly support the latter hypothesis. Studies with RyRs incorporated into planar lipid bilayers suggest that CaMK phosphorylation of RyR increases the sensitivity of release to Ca^2+ ^and thus increases RyR open probability [63]. We implemented CaMK modulation of the RyR in an analogous way to LCC. That is, we made parameter , which defines the Ca^2+ ^dependent transition rate from open state 1 to open state 2 (see Figure 1 in the original publication of Keizer et al. [64]), CaMK-dependent as described by the below equation.

(5)

This mechanism increases RyR open probability as a function of increasing CaMK activity. The maximal CaMK-dependent increase of *J*_*rel *_was set to ~10%.

### The Transgenic Myocyte Models

In this study, we evaluate three transgenic models: 1) the PLB knockout, 2) the disrupted CaMK-dependent modulation of LCC, and 3) the model of three-time overexpression of CaMK. To carry out the first test case we implemented two versions of the model according to the work by Li et al. [19]. Both of them lack the PLB inhibition of SERCA, i.e. we set value of parameter CaMK_reg to 1 in the denominator of equation (3). In addition to this, the first version also has a reduced RyR expression level (by 25%) as reported by Chu et al. [33]. The second version describes a theoretical situation, where there are no such compensations. We refer to these two model versions in the text as "PLB-KO" and "PLB-KO*", respectively.

The two versions of the second test case were implemented by setting the value of parameter *CaMK_reg *in equation (4) either to 0 or 1. That is, the first version of this model refers to a knock-out of CaMK-dependent phosphorylation of LCC and the second version to a constitutively active CaMK phosphorylation of LCC. These transgenic models have not been studied experimentally; instead they have been formulated according to the work of Grueter et al. [65] and Wu et al. [87], respectively. We refer to these two model versions in the text as "bLCC" and "cLCC", respectively. For the study of auto-regulatory link between LCC and RyR, we also estimated how the L-type calcium current changes in an acute disruption of the CaMK-dependent regulation of LCC. That is, we calculated what the I_Ca, L _would be if everything else remained normal. These simulations thus correspond to "WT clamp" protocol that cannot be reproduced *in vivo*.

The third test case demonstrates the effects of threefold overexpression of CaMK corresponding to the transgenic mouse model of heart failure developed by Maier et al. [20]. We implemented the overexpression by increasing the total values of CaMK concentrations from 10 μM to 30 μM (see Table 4 for details). Furthermore, we made a second model version of this test case that includes also the compensatory changes reported by Maier et al. [20]. That is, we changed the expression levels of SERCA, RyR and NCX to 68%, 42% and 210%, respectively, compared to WT, and we also reduced the SERCA/PLB ratio by 17% [20]. These two model versions are referred in the text as "CaMK3X*" and "CaMK3X", respectively. Additionally, we implemented parallel versions of the three test cases that include the putative CaMK induced diastolic SR leak according to the following equation:

(6)

This description of the leak sets, for example in the CaMK3X test case, about half a percent of the RyR channels to a constantly open state during diastole at 1 Hz pacing.

### Simulation Protocols

The presented mathematical model is a set of 91 ordinary differential equations (ODEs). It was implemented to the Matlab™ environment of technical programming. Simulation results were obtained by numerically integrating the model equations with a stiff ODE solver method (ode15s). Unless stated otherwise, all the presented results are steady state results that were obtained by letting simulations run as long as was needed for the model to reach a pacing steady state. The normal stimulus that we used was a current pulse (amplitude -80 pA/pF and length 0.5 ms) that was repeated according to the pacing frequency.

The caffeine pulse experiment was executed *in silico *by setting SERCA closed and RyR constantly open. The open probability was set to 0.25 instead of 1 to reflect the fact that in experiments the application of caffeine does not result in simultaneous and infinitely fast opening of RyR channels. The Ca^2+ ^released from the SR is thus extruded from the cell mainly by NCX and secondly by PMCA (see Figure 2E).

### Sodium-Calcium Exchanger

The description of the NCX current (*I*_*NCX*_) in the Bondarenko model [53] is based on a combined chemical reaction and free-energy barrier model developed by Luo and Rudy [55].

(7)

Bondarenko et al. used the cytosolic [Ca^2+^]_i _value in the fourth term of the equation. However, we modified the equation according to previous models [56,78], i.e., we upscaled the [Ca^2+^]_i _with a constant *f*_*NCX*_. This simulates the higher sub-sarcolemmal [Ca^2+^]. After iteration, we obtained the best physiological fit when the *f*_*NCX *_value was set to 2. To adjust the Ca^2+ ^recirculation fraction to a physiological level (see Table 2), we reduced the scaling factor for the total current *k*_*NCX *_to 125 pA/pF.

### Contractile Element

The contractile element of the model is based on the E-C coupling model published by Cortassa et al. [54]. That is, we implemented equations A81 and A96-121 to our model. Since the Cortassa model is based on guinea pig data, we adjusted some of the parameters related to the myofilament Ca^2+ ^sensitivity to obtain Ca^2+ ^transient and contraction force characteristics (force at diastolic [Ca^2+^], maximum amplitude of the force, and time-to-peak of the force) that are typical for mouse cardiac myocytes [21,22]. Parameter modifications are presented in Table 3.

### Authors' contributions

JTK implemented the regulatory mechanisms of the model, carried out the *in silico *experiments and data analysis and drafted the manuscript. TK implemented the electrophysiology part of the model and helped to draft the manuscript. JT implemented the contractile element part of the model. MW participated in the design and coordination of the study and helped to draft the manuscript. PT designed the study and drafted the manuscript. All authors read and approved the final manuscript.
